# The study of JAZF1-mediated apoptosis of decidual stromal cells by activating the NF-κB signaling pathway in spontaneous preterm birth

**DOI:** 10.1186/s12884-025-07983-5

**Published:** 2025-08-27

**Authors:** Mei-Qin Gong, Ya-Yi Hu, Yong-Qing Zhang, Xiao-Dong Wang

**Affiliations:** 1 Department of Obstetrics and Gynecology, West China Second University Hospital, Sichuan University, Chengdu, China; 2 School of Computer Science, Chengdu University of Information Technology, Chengdu, China

**Keywords:** Juxtaposed with another zinc finger gene 1, Spontaneous preterm, Decidual stromal cells, NF-κB, COX-2

## Abstract

**Purpose:**

Juxtaposed with another zinc finger gene 1 (JAZF1) is known to be involved in various biological processes, including gluconeogenesis, insulin sensitivity, cell differentiation, lipid metabolism, and inflammation. However, its role in spontaneous preterm birth (SPTB) remains unclear.

**Patients and methods:**

We investigated the expression of JAZF1 mRNA using quantitative reverse transcription-PCR (qRT-PCR) in the decidual tissue of patients with SPTB compared to non-spontaneous preterm birth(non-SPTB). We also utilized Western blotting and ELISA to assess JAZF1 expression in peripheral blood from SPTB and non-SPTB patients. To further explore JAZF1’s role, we validated the findings by constructing both knockdown and overexpression cellular models. The apoptosis level of decidual cells was detected by flow cytometry, and Western blotting was used to measure the expression of BCL-2, BAX, IκBα, phosphorylated IκBα, and COX-2. All collected data were statistically analyzed using GraphPad Prism 9.0. Gestational age was determined by crown-rump length (CRL) measured at 11–13^+6^ weeks. We compared the means between the two sample groups using either the independent sample t-test or the Wilcoxon rank-sum test, depending on the data distribution.

**Results:**

The JAZF1 expression in peripheral blood and decidual tissue of SPTB patients was significantly lower than that in non-SPTB patients. Moreover, the level of apoptosis in decidual stromal cells was notably higher in SPTB. JAZF1 knockdown significantly reduced decidual stromal cells’ migration and invasion capabilities while promoting apoptosis, additionally, knocking down JAZF1 in decidual stromal cells elevated Bax expression and the phosphorylation of IκBα, decreasing BCL-2 expression, further promotes the release of factors associated with uterine contractions, while contrasting outcomes were observed in the overexpression experiment.

**Conclusion:**

The identification of JAZF1 as a reliable molecular marker for spontaneous preterm labor offers novel insights for developing early warning biomarkers or targeted therapies.

**Supplementary Information:**

The online version contains supplementary material available at 10.1186/s12884-025-07983-5.

## Introduction

Preterm birth(PTB) is defined by the World Health Organization (WHO) as the delivery that occurs before 37 weeks of pregnancy or fewer than 259 days [1]. PTB increases the risk of cardiovascular disease and metabolic syndrome in adulthood [2]. About 15 million babies are born preterm annually, with an increasing trend in cases rates worldwide, putting the global PTB rate at 11% [3]. PTB is classified into SPTB and iatrogenic preterm birth. SPTB defined as preterm delivery without medical intervention-including preterm premature rupture of membranes (PPROM)-represents the most prevalent subtype. As a complex, highly heterogeneous syndrome, PTB arises from multiple risk factors and etiologically-linked conditions that drive distinct phenotypic outcomes across pregnancy, postnatal, and early childhood stages [4]. Some researchers provided an update and add new elements that have emerged in the last decade to promote an etiologically-based PTB taxonomy in epidemiological, clinical, and research activities [5].

PTB shares many pathways with term birth, and it is thought to result from the early activation of these pathways [6]. The release of factors associated with uterine contractions, such as cyclooxygenase type 2 (COX-2) and prostaglandin F2α(PGF2α), plays a significant role in inducing labor [7].

Decidual stromal cells-the principal cellular constituent of decidual tissue-maintain normal gestation by secreting diverse inflammatory and chemotactic factors that promote immune cell-trophoblast cross-talk [8]. It has been shown that endometrial stromal cells undergo apoptosis in an autocrine or paracrine manner during the process of decidualization, and that appropriate apoptosis contributes to the establishment of decidualization and the maintenance of decidualization homeostasis [9–11]. Disruption of this balance can lead to various pathological conditions, including PTB and spontaneous abortion. The decidua is an important link in the “labor cascade”, therefore, if the secretion and coordination of the decidua are disturbed, the process of labor initiation will be advanced or impeded [12].

It has been noted explicitly that an increase in decidual stromal cell apoptosis plays a critical role in SPTB [13]. Various protein molecules, such as those from the BCL-2、BAX and caspase families, are involved in the apoptotic processes of these cells [14]. It has been found that abnormally elevated BAX levels cause recurrent miscarriages by inducing apoptosis [15, 16], however, the regulatory mechanisms remain unclear and warrant further investigation.

JAZF1 also known as TIP27, was first identified as a novel TAK1-interacting protein in 2004 [17]. JAZF1 acts as a transcriptional co-regulator that interacts with a range of nuclear receptors and protein kinases involved in cellular energy metabolism [17]. Previous studies have shown that JAZF1 is involved in gluconeogenesis, insulin sensitivity, cell differentiation, lipid metabolism, and inflammation [18, 19]. The JAZF1 gene is widely expressed in a variety of tissues including liver, fat, skeletal muscle and pancreas [17, 20]. Under specific pathological conditions, downregulation of JAZF1 expression is observed, and this reduction in JAZF1 levels has been implicated in the pathogenesis of metabolic disorders such as obesity, diabetes, and hepatic steatosis [21–24]. Currently, the only study analyzing the association between JAZF1 gene polymorphisms and unexplained recurrent miscarriage in Han Chinese couples was published in 2015 by Professor Chen Zijiang’s team, both domestically and internationally [25]. Additionally, overexpression of JAZF1 in primary cardiac microvascular endothelial cells (CMECs) reduced the phosphorylation level of Forkhead box protein O1 (FOXO1), while downregulated phosphorylation of cAMP-responsive element-binding protein (CREB) was observed in hepatocytes of JAZF1 transgenic (JAZF1-Tg) mice, collectively suppressing apoptosis in these cell types [26, 27]. Notably, FOXO1 and CREB are key regulators of decidualization [28]. Based on these findings, we hypothesize that JAZF1 may play a critical role in maintaining decidualization homeostasis and supporting pregnancy progression.

Furthermore, researches have indicated JAZF1 inhibits proliferation and induces apoptosis in papillary thyroid cancer cells by inhibiting activation of the TAK1/NF-κB signaling pathway [29, 30]. As a key point for various transduction pathways within the cell, NF-κB regulates multiple physiological activities, including inflammation and apoptosis, and is involved in disease development [31]. When cells experience inflammation, IκB undergoes phosphorylation and is then degraded by proteases. This degradation leads to the translocation of NF-κBp65 from the cytoplasm to the nucleus, activating the NF-κB signaling pathway, which influences the expression of BCL-2 proteins and subsequently controls the apoptotic process [32].

Therefore, we propose the hypothesis that low expression of JAZF1 may mediate apoptosis in decidual cells in SPTB through activation of the NF-κB signaling pathway.

## Materials and methods

### Decidual tissue specimens and peripheral blood

Human decidual tissue samples and maternal peripheral blood were collected for total 60 samples at West China Second Hospital, Sichuan University, between 2022 and 2024. The enrollment criteria were as follows: (1) gestational age of 28–36^+6^ weeks; (2) SPTB; (3) single pregnancy. The diagnostic criteria for preterm birth and spontaneous preterm birth were based on the Clinical Guidelines for Prevention and Management of Preterm Birth issued by the Chinese Medical Association. Pregnant women with any of the following obstetric complications or comorbidities were excluded: Hypertensive disorders of pregnancy (including gestational hypertension and preeclampsia); Intrahepatic cholestasis of pregnancy(ICP); Polyhydramnios; Uterine malformations; Cervical insufficiency; Placental abruption; Acute or chronic inflammatory conditions, patients with confirmed infections, such as those presenting with maternal fever, leukocytosis, and marked uterine tenderness were excluded. In the control group, patients underwent cesarean section resulting from iatrogenic cesarean delivery indicated for maternal or fetal conditions, such as maternal cardiac disease, vasa previa, or fetal arrhythmias. Fresh decidual tissue from placental attachment sites-30 SPTB and 30 non-SPTB cases-underwent normal saline washing, part of which were stored in a freezer at −80℃, and the other part were fixed with formaldehyde and stored in a freezer at 4℃. This study adhered to the principles of the Helsinki Declaration and was approved by the Ethics Committee of West China Second Hospital, Sichuan University. All participants provided written informed consent for using their samples in the study.

### Induced decidualization of human immortalized endometrial stromal cells

The human immortalized endometrial stromal cell line (HESC, CRL-4003) was obtained from Shanghai Jitai Biological Co.,Ltd. The cells were cultivated in Dulbecco’s Modified Eagle Medium (DMEM) supplemented with 10% fetal calf serum in a 5% CO_2_ atmosphere at 37 °C. The specific methods of decidualization: (1) Add methoxyprogesterone to 4µM and dibutyl cyclophosphamide to 1 mM in HESC cell culture system and then incubate. (2) Cells were cultured at 37℃ with 5% CO_2_, and the medium was changed after 2 days. (3) Cells were collected on days 0, 1 and 2 after the addition of medroxyprogesterone and dibutyl cyclophosphamide, respectively, and the effect of HESC cells decidualization were confirmed by using Western Blotting assay for the decidual markers IGFPB1 and FOXO1.

### Constructing decidualized HESCs with JAZF1 knockdown and JAZF1 overexpression

The specific scheme was as follows: (1) Cell spreading: Collect decidualized HESC cells and spread them in 6-well plates at a cell density of 5 × 10^5^, and incubate them overnight at 37℃ in a cell culture incubator. (2) The next day, the cell morphology was observed and the old medium was aspirated away, 25 µl of DMEM medium containing siRNA (General Biologicals, China) was added to each well to constructing Decidualized HESCs with JAZF1 Knockdown. In this step, if we wanted to constructing Decidualized HESCs with JAZF1 overexpression, we added 25 µl of DMEM medium containing the overexpression plasmid (Cloud-Clone Corp., China) to each well. The transfection efficiency was increased with Lipofectamine 3000 (Life Technologies, USA) according to the manufacturer’s protocol using Lipofectamine 3000 (Life Technologies, USA) according to the manufacturer’s protocol. (3)Western Blotting and RT-qPCR were used to confirm the transfection efficiency of JAZF1 at 48 h after transfection. Cell numbers were quantified as cells per chamber.

### RNA extraction and qRT-PCR analysis

Total RNA was isolated from maternal decidual tissue in the experimental and control groups using Trizol reagent (Invitrogen, USA), following the manufacturer’s protocol. The RT Easy™ II Reverse Transcription Kit (FOREGENE, Chengdu, China) was then utilized to reverse transcribe the total RNA into cDNA. GAPDH served as the endogenous control. The expression level of JAZF1 was analyzed using the 2^−△△Ct^ method. The primers used for qRT-PCR were as follows:

- JAZF1:
- Forward: 5’-ATCGAGCACATCGAGGACAAC-3’.
- Reverse: 5’-GAGCTTCGGCTGAATCTTCTT-3’.
- GAPDH:
- Forward: 5’-GGAGCGAGATCCCTCCAAAAT-3’.
- Reverse: 5’-GGCTGTTGTCATACTTCTCATGG-3’.

### Western blotting

Western blotting analysis was conducted as previously described [33]. The primary antibodies used in the experiments included anti-JAZF1 from Invitrogen (USA), and anti-BCL-2, anti-β-actin, anti-GAPDH, anti-BAX, anti-IGFBP1, anti-FOXO1, anti-IκBα, anti-PI3Kα, anti-P65, anti-PP65, and anti-COX2 from Wuhan Sanying Biotechnology Co., Ltd. Secondary antibodies included goat anti-rabbit and goat anti-mouse.

### ELISA assay

Collect 5 ml of patient’s peripheral blood, centrifuge at 3000 rpm for 10 min at 4℃. Preserve the supernatant according to the manufacturer’s instructions and test for JAZF1 content using an ELISA kit, and ELISA kits were also used to measure the levels of prolactin (PRL), prostaglandins (PGs), IL-8, and TNF-α in the cell culture medium supernatants, which were also collected and centrifuged from patient’s peripheral blood. Measure the absorbance at 450 nm with an enzyme meter.

### EdU assay

Collect 80–90% growth of decidualized HESC cells, spread them in 96-well plates at a density of 5000/well, and incubate them overnight in an incubator. Cell proliferation assay was performed according to the Cell-Light EdU Apollo488 kit. Fresh medium containing 50µM EdU (1:1000 dilution) per well was incubated in a humidified incubator at 37℃ with 5% CO_2_ and protected from light for 2–4 h. A blank background control group without EdU was set up at the same time. Take pictures under fluorescence microscope for preservation, and use Image J software for counting.

### Colony formation assay

Collect metamorphosed HESC grown to 80–90% and inoculate them at a density of 500/well in a 6-well plate. Then incubate at 37℃ for 24 h. After about 14 days, colonies were fixed with methanol and stained with 0.5% crystal violet for 30 min at room temperature. The number of colonies was counted and photographed to assess the proliferative capacity of decidualized HESC cells.

### Transwell assay

We used Transwell chambers for invasion experiments: 100 µl of 300 µg/ml matrix gel was first applied to the upper surface of the polycarbonate membrane, which was then placed in the chambers at 37℃ for 30 min. Collection of decidualized HESC grown to 80–90%, inoculation of 1 × 10^5^ decidualized HESC in each upper chamber and addition of medium. Subsequently add 600 µl of medium containing 20% FBS to each lower chamber and incubate for 24 h. The invaded cells were fixed with methanol and stained with 0.1% crystal violet for 10 min at room temperature. Photographs were taken under the microscope for preservation, and the images were processed and counted using Image J software. For the Transwell migration assay, we inoculated 1 × 10^5^ decidualized HESC into the upper chamber of a Transwell that did not contain matrix gel, followed by the same steps as above.

### Flow cytometry assay

Place 1 × 10⁶ cells in a flow cytometry tube, centrifuge at 1200 rpm for 3 min, and discard supernatant. Prepare reagents according to the manufacturer’s instructions of the FITC Annexin V Apoptosis Detection Kit. Resuspend cell pellet in 100 µl 1×Binding Buffer. Add 1 µl FITC Annexin V staining solution. Incubate for 10 min at RT in the dark with gentle agitation. Add 1 µl propidium iodide (PI) solution. Subsequently, the cells were stained with PI at 4℃. Then, the cells were detected using a flow cytometer.

### Statistical analysis

All data from this experiment were statistically analyzed using GraphPad Prism 9.0 software. According to the data distribution, the mean differences between the two sample groups were compared using the independent samples t-test or the Wilcoxon rank-sum test. For repeated measurements involving a single factor, one-way ANOVA was employed, while two-way ANOVA was utilized for multiple factors. Statistical significance was defined as *p < 0.05; **p < 0.01; ***p < 0.001; ns, *p*>0.05. All data are shown as the mean ± SD (*n* = 3).

## Results

### JAZF1 is significantly down-regulated in spontaneous preterm birth

**Fig. 1:**
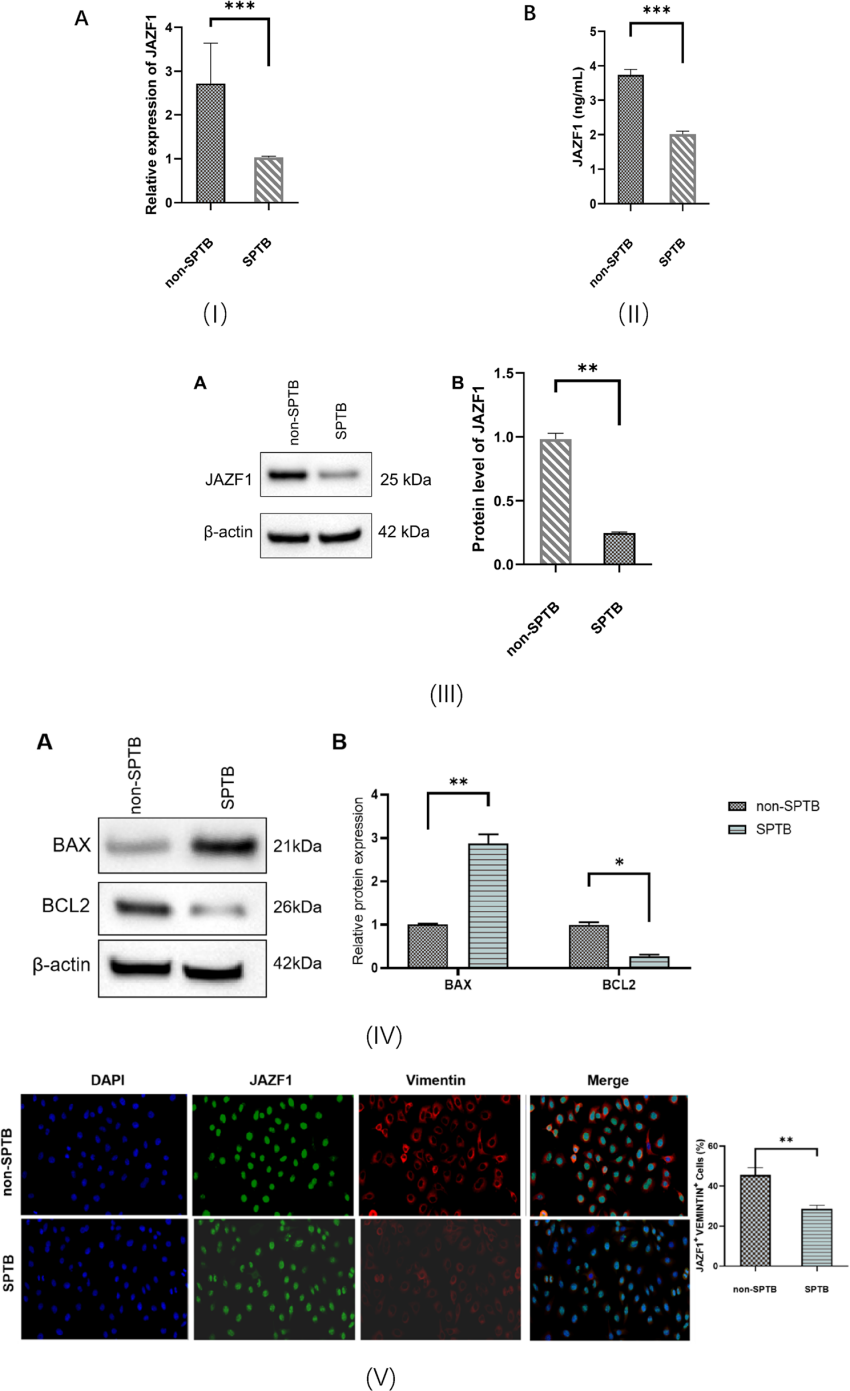
(**I**), qRT-PCR analysis of JAZF1 mRNA levels in decidual tissues between the two groups; (**II**), ELISA quantification of JAZF1 protein expression in peripheral blood of both groups; (**III**), Western blotting of JAZF1 protein levels in decidual tissues from the two groups; (**IV**), Western blotting analysis of apoptosis-related markers in decidual tissues of both groups, including the pro-apoptotic protein BAX and anti-apoptotic protein BCL-2; (**V**), Immunofluorescence staining showing the subcellular localization of JAZF1 in decidual cells (blue: DAPI-stained nuclei; green: JAZF1 signal; scale bar: 20 μm).*p < 0.05; **p < 0.01; ***p < 0.001

To assess the expression of JAZF1 mRNA in maternal decidual tissues, we conducted qRT-PCR, and ELISA assay was also performed for 60 samples. Additionally, we performed Western blotting to evaluate JAZF1 expression in decidual tissues. To explore the relationship between decidual cell apoptosis and SPTB, we conducted a Western blotting assay to detect apoptosis-related proteins BAX and BCL-2 expression levels in decidual tissues. The localization of JAZF1 in cells was determined through immunofluorescence staining. As illustrated in Fig. 1, JAZF1 immunofluorescence was primarily observed in the cytoplasm. Our analysis revealed that JAZF1 was significantly down-regulated in the decidual tissues of the SPTB group compared to the non-SPTB group. Similarly, the mRNA expression level of JAZF1 in maternal peripheral blood was significantly lower in the SPTB group than in the non-SPTB group. The findings demonstrated that downregulation of JAZF1 expression promoted SPTB initiation, potentially mediated through its apoptosis-inducing effects in cells.

### The effect of knockdown or overexpression of JAZF1 in proliferation, migration, and invasion of decidualized HESCs

**Fig. 2:**
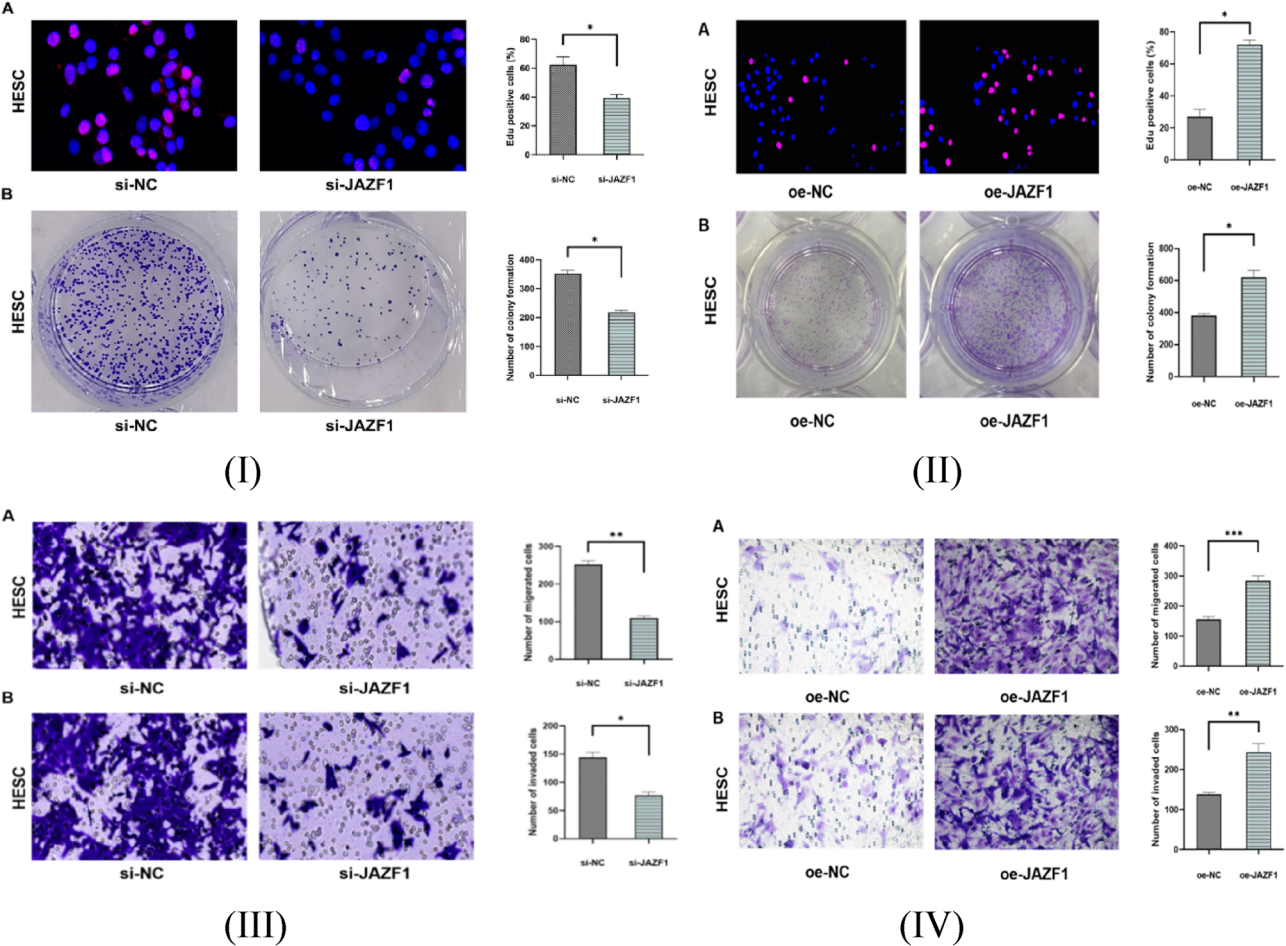
(**I**),Proliferation analysis: (A), EdU assay showing synthesis activity in decidualized HESCs; (**II**),100×enlarged images; (B), Colony formation assay quantifying clonogenic capacity of decidualized HESCs; (**III**)(**IV**), Migration and invasion assays: Transwell chamber assay assessing cellular migration (uncoated membrane) and invasion (Matrigel-coated membrane); si-JAZF1,JAZF1 knockdown group; si-NC, Negative control group in the knockdown experiment; oe-JAZF1,JAZF1 overexpression group; oe-NC, Negative control group in the overexpression experiment; *p < 0.05; **p < 0.01;***p < 0.001

We performed EdU and colony formation assays to verify whether JAZF1 knockdown or JAZF1 overexpression affects the proliferation of decidualized HESCs. As shown in Fig. 2(I)(II), both Edu assay and cloning assay showed that JAZF1 knockdown reduced cell proliferation of decidualized HESCs, opposing trends were detected in overexpression assays. Next, we performed a Transwell assay to verify whether JAZF1 knockdown or JAZF1 overexpress affects the migration and invasion of decidualized HESCs. As shown in Fig. 2(III)(IV), the Transwell assay showed that JAZF1 knockdown reduced the migration and invasion of decidualized HESCs, the overexpression experiments yielded opposing results.

### The effect of knockdown and overexpression of JAZF1 in apoptosis of decidualized HESCs

**Fig. 3:**
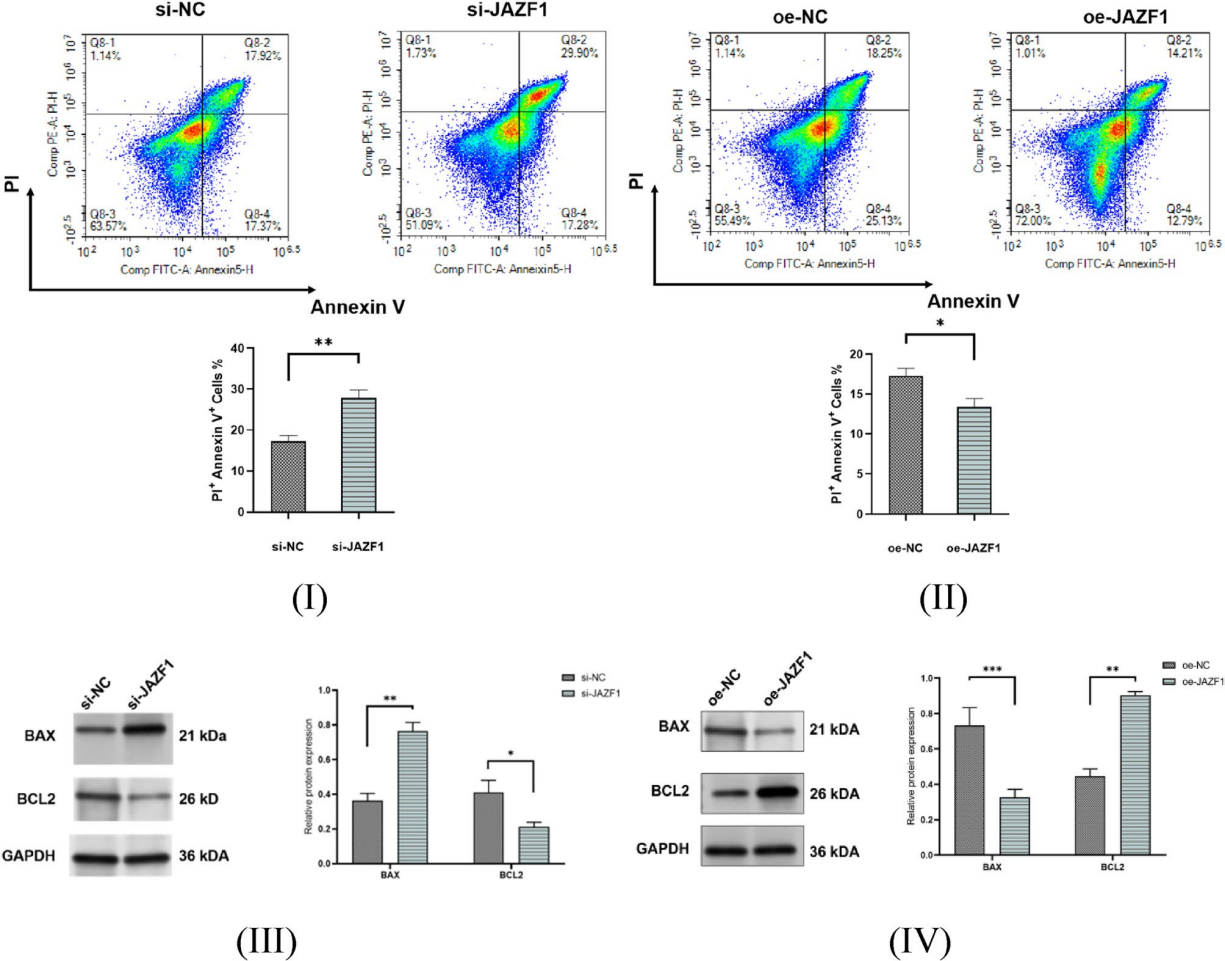
Flow cytometry analysis apoptosis of Decidualized HESCs. (**I**)(**II)**, HESCs apoptosis levels in si-JAZF1 and oe-JAZF1 groups; (**III**)(**IV**), Western blotting analysis was performed to quantify the protein levels of BAX (pro-apoptotic) and BCL-2 (anti-apoptotic) in si-JAZF1 and oe-JAZF1 groups; Normalized to GAPDH expression.*p < 0.05; **p < 0.01;***p < 0.001

We constructed models for JAZF1 knockdown and JAZF1 overexpression in decidualized HESCs, measurement of apoptosis levels in HESCs after JAZF1 knockdown and overexpression using flow cytometry. As shown in Fig. 3(I)(II), knockdown of JAZF1 increased apoptosis in HESCs, whereas JAZF1 overexpression decreased apoptosis.

We found that the expression of the pro-apoptotic protein BAX increased assessed using Western blotting, the expression of the anti-apoptotic protein BCL-2 decreased in the si-JAZF1 group compared to the si-NC group, while contrasting outcomes were observed in the overexpression experiment, as shown in Fig. 3(III)(IV).

### The impact of JAZF1 on the NF-κB signaling pathway

**Fig. 4:**
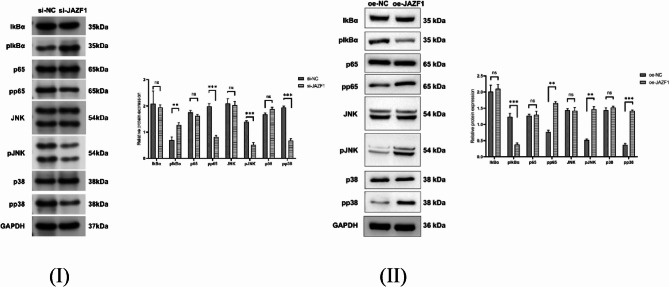
(**I**)(**II)**, Protein levels of IκBα, phosphorylated p65, JNK, and p38 were assessed by Western blotting in si-JAZF1 and oe-JAZF1 groups; With GAPDH as the loading control. **p < 0.01; ***p < 0.001; ns, *p*>0.05

The NF-κB and MAPK pathways are known to play essential roles in regulating apoptosis. Increased phosphorylation of IκBα is a marker of NF-κB pathway activation, whereas the phosphorylation of JNK and p38 indicates MAPK pathway activation. We assessed the expression and phosphorylation of proteins related to the NF-κB and MAPK pathways in si-JAZF1 and oe-JAZF1 groups. The results indicated that JAZF1 knockdown significantly increased the phosphorylation of IκBα while decreasing the phosphorylation of p65, JNK, and p38, while contrasting outcomes were observed in the overexpression experiment, as depicted in Fig. 4(I)(II).

### The COX-2 expression and PGs secretion of JAZF1 knockdown and overexpress in decidualized HESCs

**Fig. 5:**
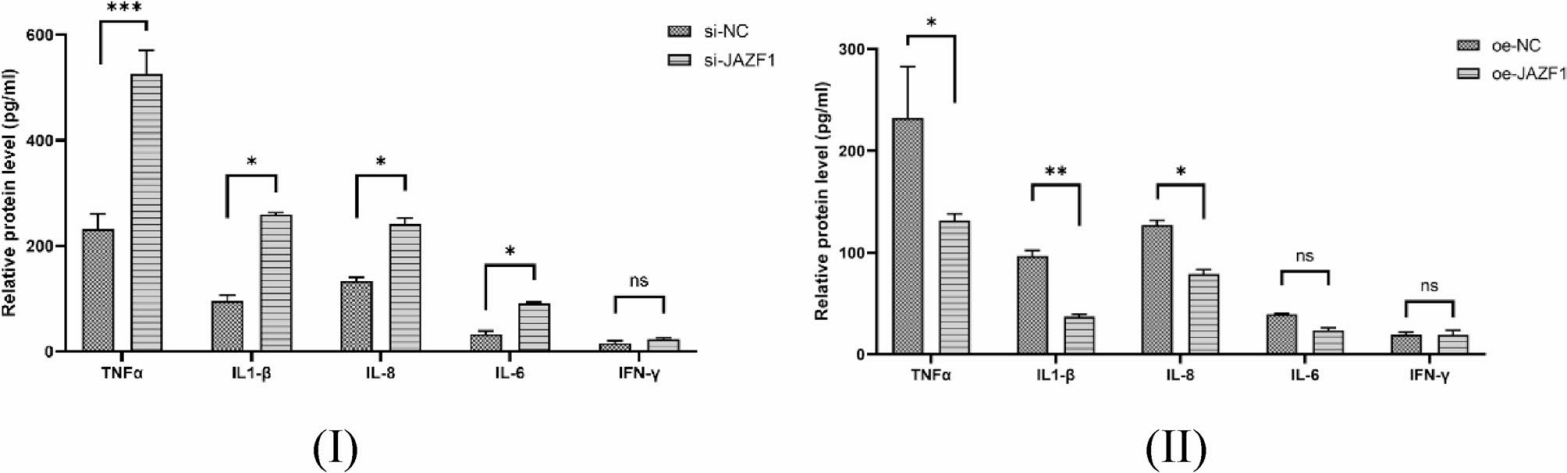
**I II**, Serum levels of TNF-α, IL-1β, IL-6, IL-8, and IFN-γ were measured using enzyme-linked immunosorbent assay (ELISA) in both si-JAZF1 and oe-NC groups; *p < 0.05; ***p < 0.001; ns, *p*>0.05

**Fig. 6:**
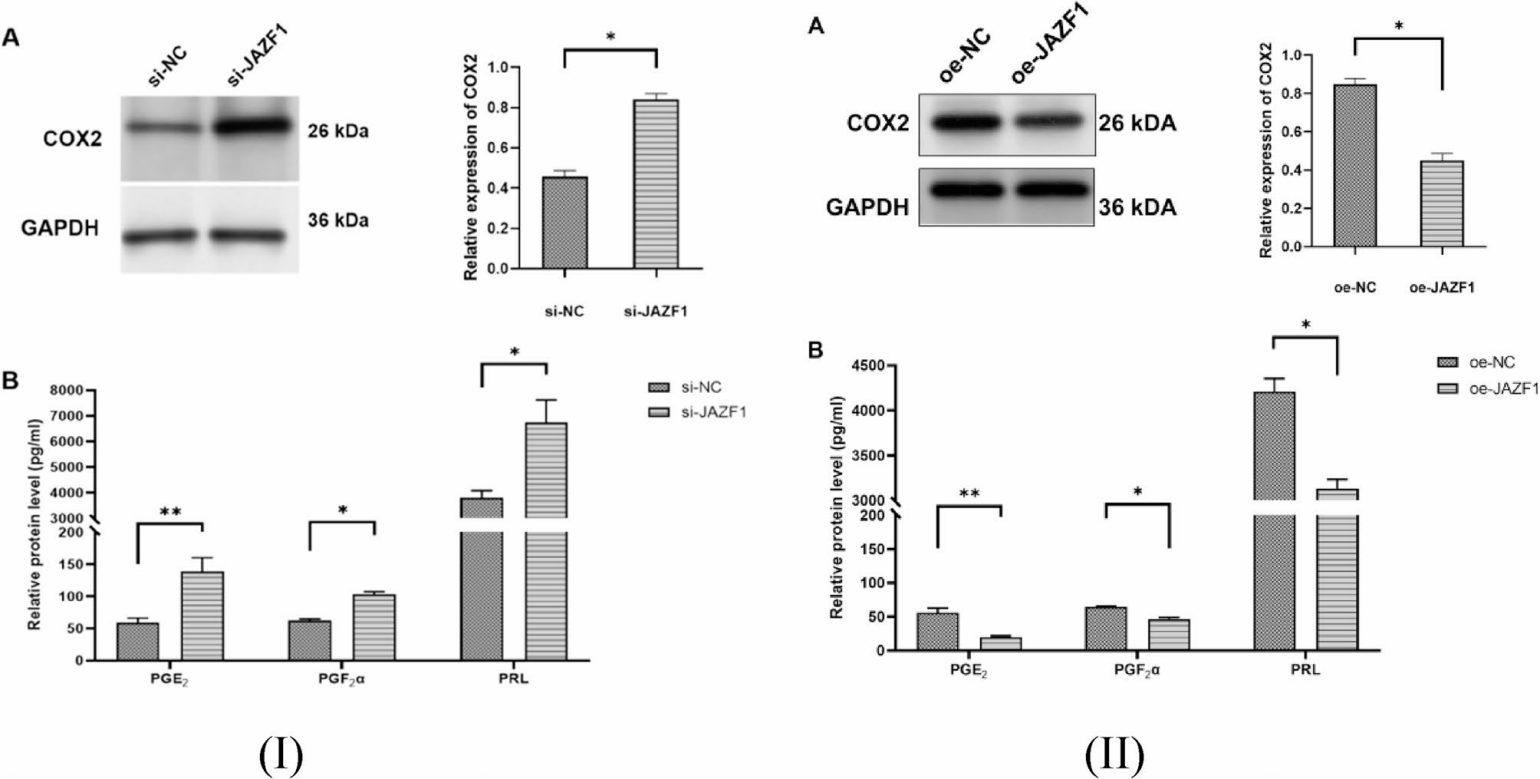
(**I**)(**II**)(A), Western blot analysis of cyclooxygenase-2 (COX-2) protein expression in si-JAZF1 and oe-JAZF1 groups; (**I**)(**II**)(B), ELISA quantification of prostaglandinE2(PGE2), prostaglandinF2α(PGF2α), and prolactin (PRL) concentrations in cell culture supernatants in both si-JAZF1 and oe-JAZF1 groups; *p < 0.05; **p < 0.01

Numerous studies have suggested that inflammation is a key mechanism in initiating human labor, whether preterm or full-term [34]. The results demonstrated a significant increase in TNF-α, IL-1β, IL-8, and IL-6 levels in the supernatant of decidualized HESCs with JAZF1 knockdown, in contrast, opposing effects were detected upon overexpress group, as shown in Fig. 5(I)(II). Inflammatory cytokines regulate the release of substances associated with uterine contraction, such as prostaglandins (PGs), which can result in uterine contractions and labor initiation [35]. We further investigated the expression of COX-2 protein and the content of PGE2 and PGF2α in JAZF1 knockdown decidualized HESCs. Our findings revealed that JAZF1 knockdown increased COX-2 protein expression, as determined through Western blotting, and enhanced the secretion of PGE2, PGF2α, and prolactin (PRL) as measured by ELISA in decidualized HESCs, while contrasting outcomes were observed in the overexpress experiment, as illustrated in Fig. 6(I)(II).

## Discussion

The pathogenesis of spontaneous preterm labor is considered to be complex, and its exact pathogenesis remains unclear, which partly explains the limited availability of targeted and effective strategies for its prevention. Most researchers recognize the role of immune inflammation in this process. As the predominant maternal decidual cell type during pregnancy, decidual stromal cells critically support embryo implantation, placentation, and fetal development via secretion of cytokines and chemokines [36–38]. However, abnormal decidual stromal cell apoptosis or senescence disrupts maternal-fetal crosstalk, contributing to adverse pregnancy outcomes [39–42]. Excessive apoptosis of decidual cells can trigger tissue necrosis, thereby compromising decidual integrity and subsequent placental dysfunction, which may ultimately contribute to preterm birth [43]. Research has shown that decidual cell apoptosis is critically involved in the onset and progression of SPTB [44], however, the exact mechanisms involved remain unclear.

Previous studies on the JAZF1 gene primarily focused on its role in diabetes and lipid metabolism [45, 46]. Recently, it has been reported that JAZF1 also regulates biological functions in human tumors, particularly in endometrial stromal sarcomas and prostate cancer [47, 48]. Only one study published in 2015 by Prof. Chen Zijiang’s team domestically and abroad analyzed the correlation between JAZF1 gene polymorphisms and unexplained recurrent miscarriage in Han Chinese couples, hence, the role of JAZF1 in decidualization and its correlation with SPTB deserves further study. However, research regarding JAZF1 and PTB has not been previously reported. Our study is the first to examine the relationship between JAZF1 and SPTB.

Our study analyzed the expression of JAZF1 mRNA and JAZF1 protein in the decidual tissues and peripheral blood of SPTB and non-SPTB groups using qRT-PCR and ELISA. We further assessed JAZF1 protein expression in the decidual tissues of the two groups using Western blotting. We found that both JAZF1 mRNA and JAZF1 protein expression were significantly decreased in the decidual tissues and peripheral blood of patients experiencing SPTB. Additionally, we observed differences in the apoptosis-related proteins BAX and BCL-2 in the decidual tissues between the two groups. The level of decidual cell apoptosis was significantly higher in the SPTB group, consistent with previously reported findings. Through constructing JAZF1 knockdown and JAZF1 overexpression models in decidualized HESCs, flow cytometric analysis revealed a marked increase in apoptotic rate in the JAZF1 knockdown group, while contrasting outcomes were observed in the JAZF1 overexpression experiment. Therefore, our experiments further demonstrate that JAZF1 may be involved in regulating apoptosis in decidual cells.

Both the NF-κB pathway and the MAPK pathway are well-established signaling pathways involved in the regulation of apoptosis. NF-κB is a well-known transcription factor that regulates the expression of numerous genes involved in immune response, inflammation, and apoptosis [49]. Under normal conditions, NF-κB is bound to its inhibitor IκBα and is located in the cytoplasm. When cells are exposed to various stimuli, IκBα undergoes phosphorylation, ubiquitination, and degradation, allowing the released NF-κB complex to translocate to the nucleus and regulate the transcription of target genes [50]. Recent studies have demonstrated that when an organism is exposed to external stimuli, the JNK/p38 MAPK signaling pathway becomes activated. This activation leads to the phosphorylation of JNK and p38 MAPK, which subsequently modulate the expression of inflammatory cytokines [51].

To investigate the specific mechanisms through which JAZF1 modulates apoptosis in decidual cells, further cell experiments were performed. In cell experiments, silencing JAZF1 with siRNA interfered with the decidualization of endometrial stromal cells, reducing their proliferation, migration, and invasion capabilities, and as anticipated, JAZF1 knockdown led to an increase in apoptosis in decidual cells, while contrasting outcomes were observed in the overexpress experiment. In this study, the phosphorylation level of IκBα was dramatically elevated in this group-a known marker of NF-κB pathway activation [52], while the phosphorylation level of JNK and p38 were decreased. The results of this study suggest that low expression of JAZF1 modulates NF-κB signaling pathway, other than MAPK signaling pathway.

Numerous studies have suggested that inflammation is a key mechanism in initiating human labor, whether preterm or full-term [34]. The results demonstrated that knockdown of JAZF1 significantly activated the NF-κB signaling pathway, which subsequently induced a marked elevation in the supernatant levels of pro-inflammatory cytokines, including TNF-α, IL-1β, IL-8, and IL-6 in decidualized HESCs, it means that JAZF1 may promote inflammatory responses through the NF-κB signaling pathway.

The final outcome of labor initiation is attributed to rupture of membranes, cervical ripening, uterine smooth muscle contractions. Our study revealed that the expression of COX-2 protein and PGF2α secretion were significantly elevated in the JAZF1 knockdown group, and decreased in JAZF1 overexpression group. Previous studies have shown that activation of NF-κB promotes COX-2 expression [53]. These findings support the hypothesis that reduced expression of JAZF1 induces decidual cell apoptosis via activation of the NF-κB signaling pathway, which could upregulate COX-2 expression and enhances PGF2α secretion.

## Conclusions

In summary, this study underscores the role of JAZF1 in SPTB. Furthermore, our findings demonstrate that low expression of JAZF1 induces apoptosis in decidual cells through activation of the NF-κB signaling pathway. This mechanism may provide a molecular basis for decidual dysfunction in SPTB. Targeting JAZF1 could present significant diagnostic and therapeutic opportunities for SPTB, and targeting NF-κB inhibitors may be able to be used in the future to treat SPTB.

## Supplementary Information

Supplementary Material 1

## Data Availability

The studies involving humans were approved by West China Second University Hospital, Sichuan University. The studies were conducted in accordance with the local legislation and institutional requirements.
