# Supplementary figures and images for: The study of JAZF1-mediated apoptosis of decidual stromal cells by activating the NF-κB signaling pathway in spontaneous preterm birth
Source: BMC Pregnancy Childbirth. 2025 Aug 27;25:891. doi: 10.1186/s12884-025-07983-5 (PMC12382285; doi:10.1186/s12884-025-07983-5)

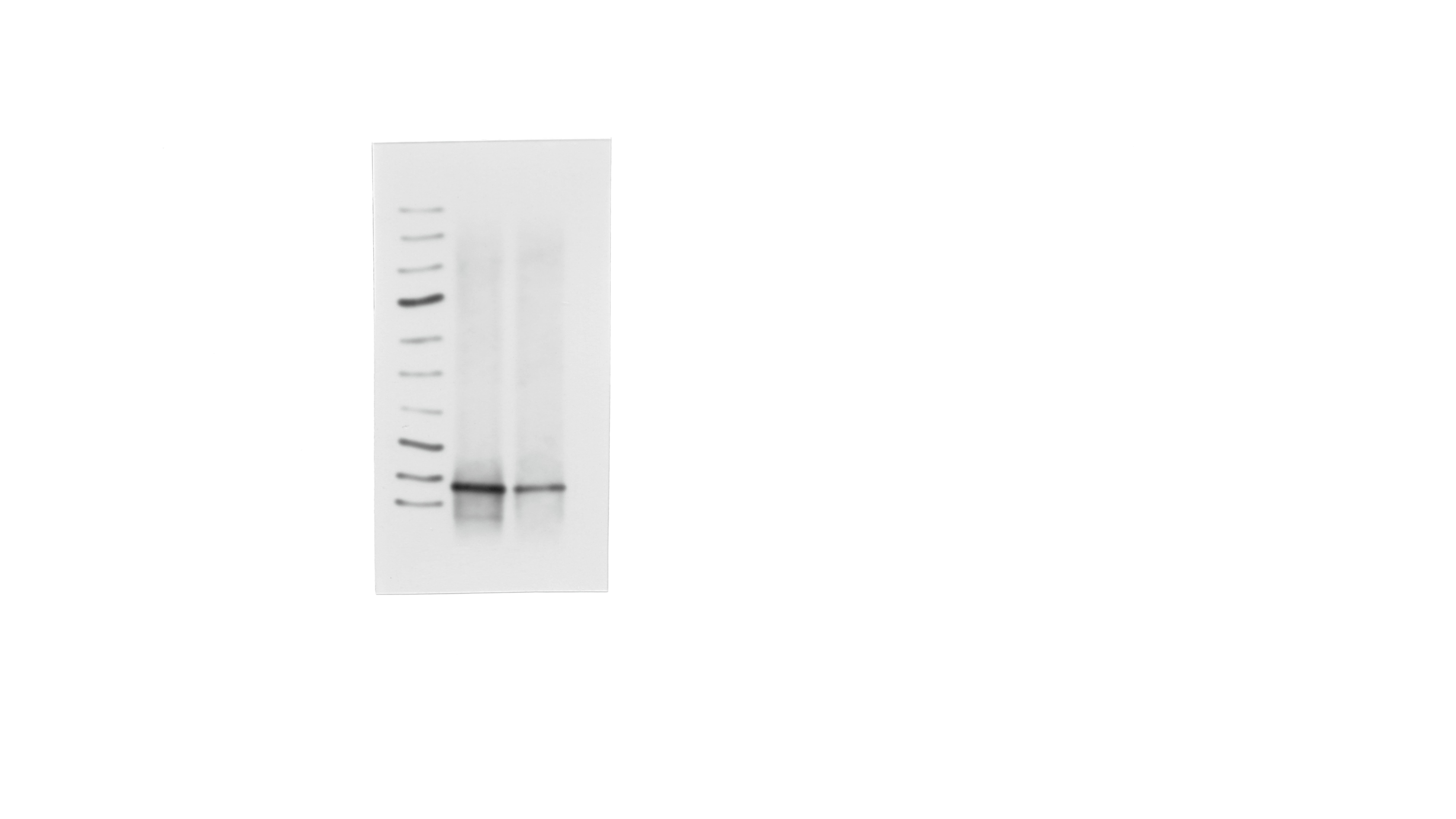

Supplement: Supplementary file 1 — Supplementary Material 1 [file 12884_2025_7983_MOESM1_ESM.zip › Overexpression experiment original WB blot/BAX BCL2 GADDH600dpi/BAX_600.tif]

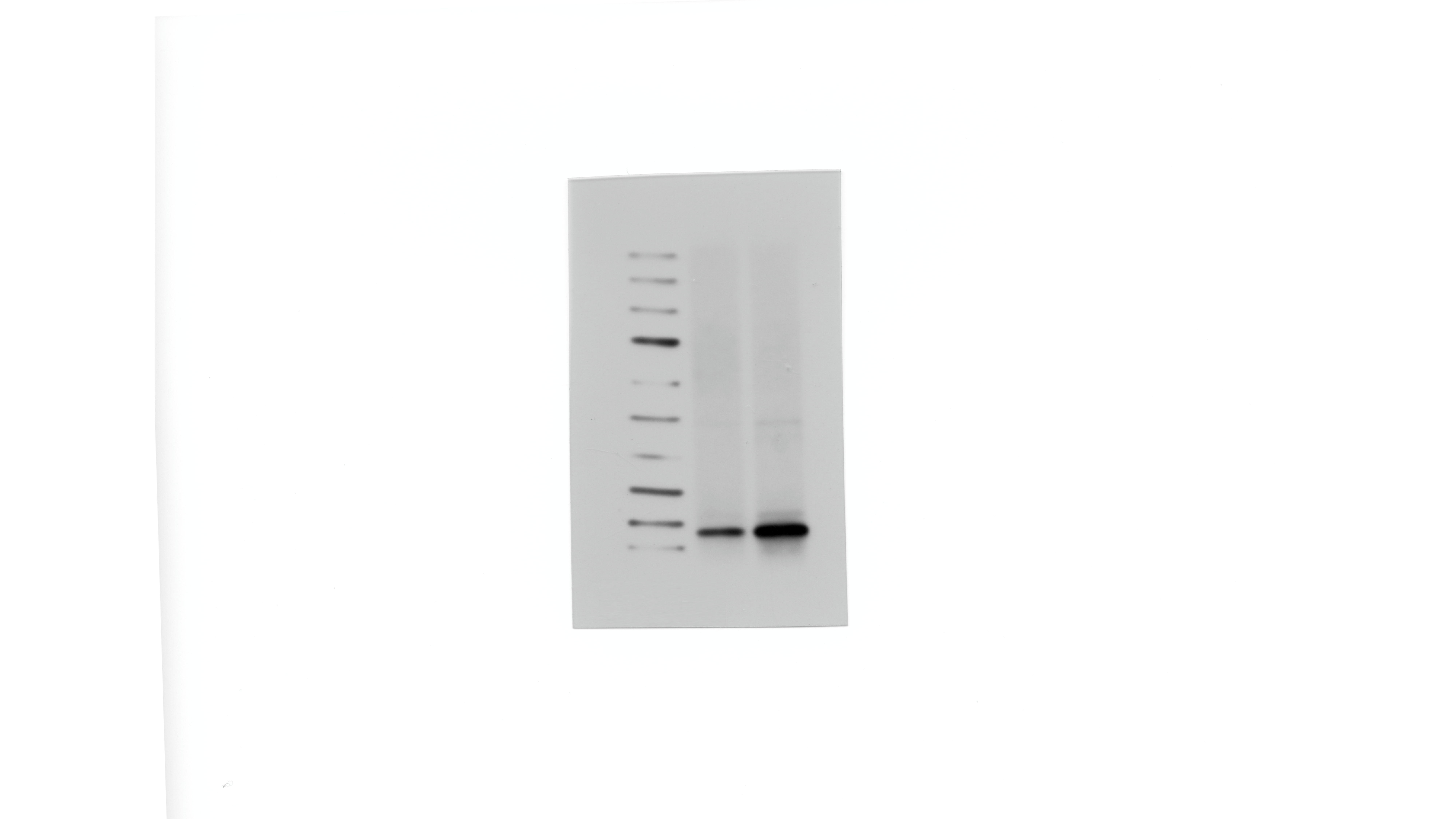

Supplement: Supplementary file 1 — Supplementary Material 1 [file 12884_2025_7983_MOESM1_ESM.zip › Overexpression experiment original WB blot/BAX BCL2 GADDH600dpi/BCL2_600.tif]

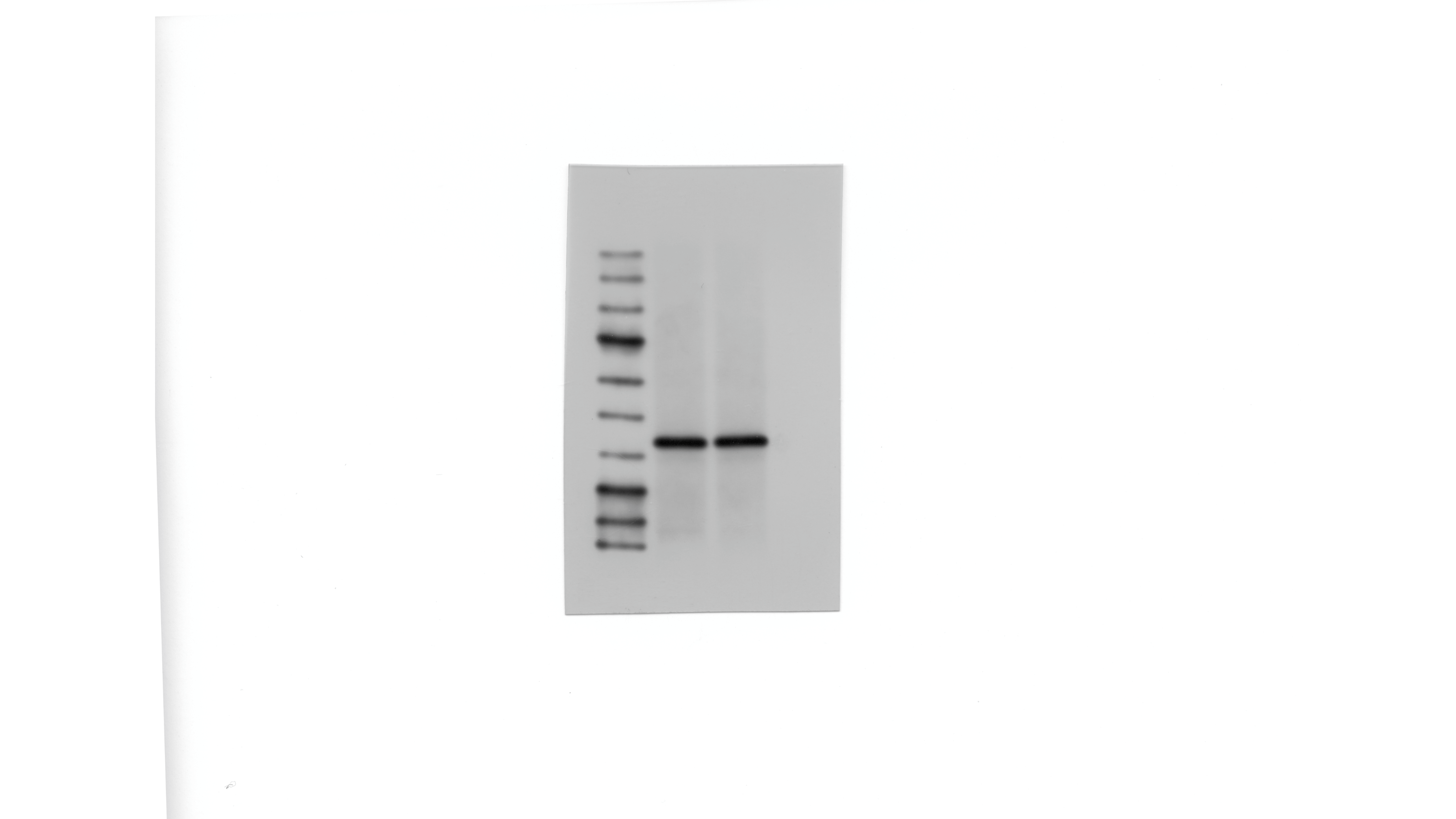

Supplement: Supplementary file 1 — Supplementary Material 1 [file 12884_2025_7983_MOESM1_ESM.zip › Overexpression experiment original WB blot/BAX BCL2 GADDH600dpi/GAPDH_600.tif]

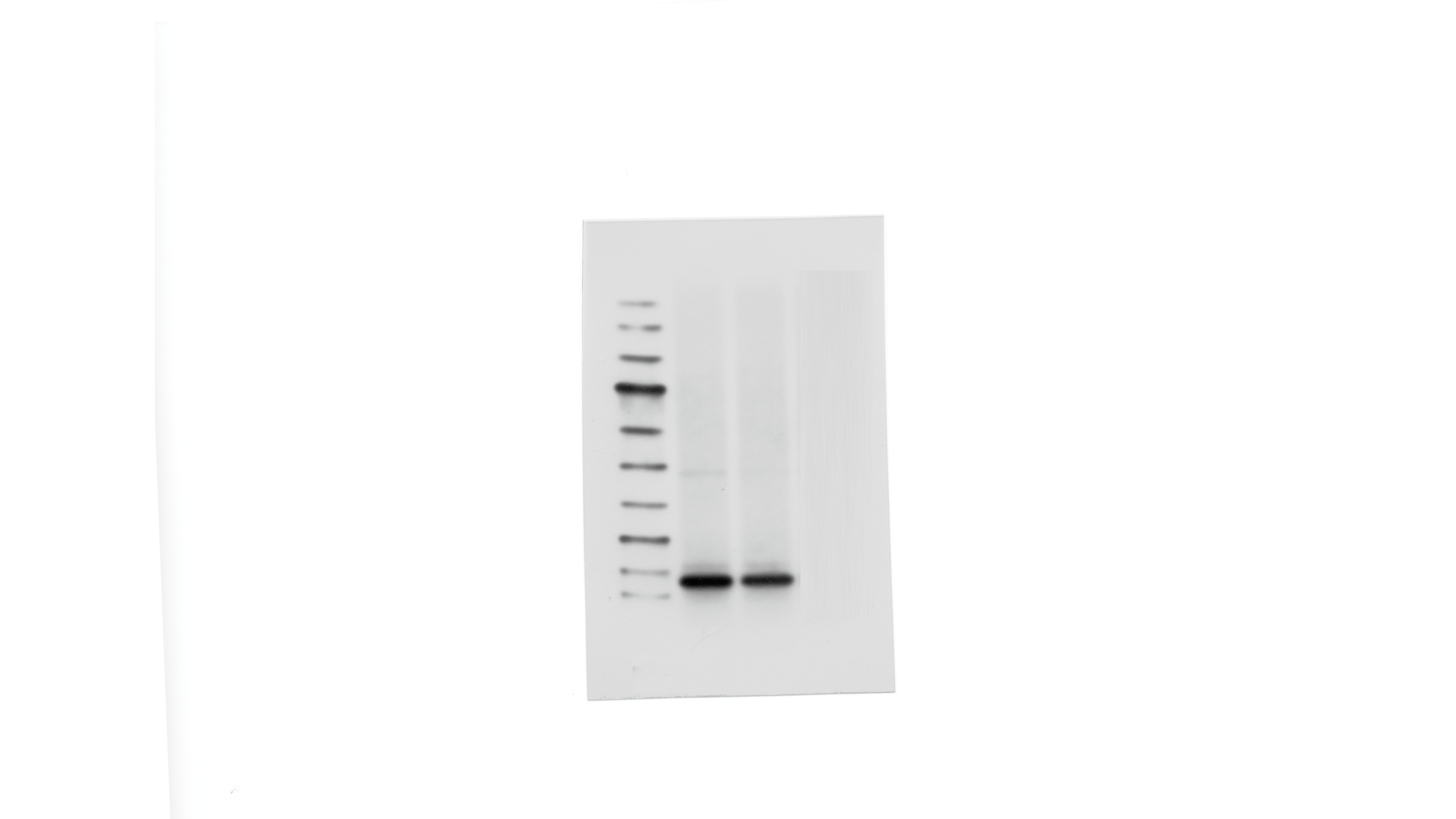

Supplement: Supplementary file 1 — Supplementary Material 1 [file 12884_2025_7983_MOESM1_ESM.zip › Overexpression experiment original WB blot/COX2 GAPDH_600dpi/COX2_600.tif]

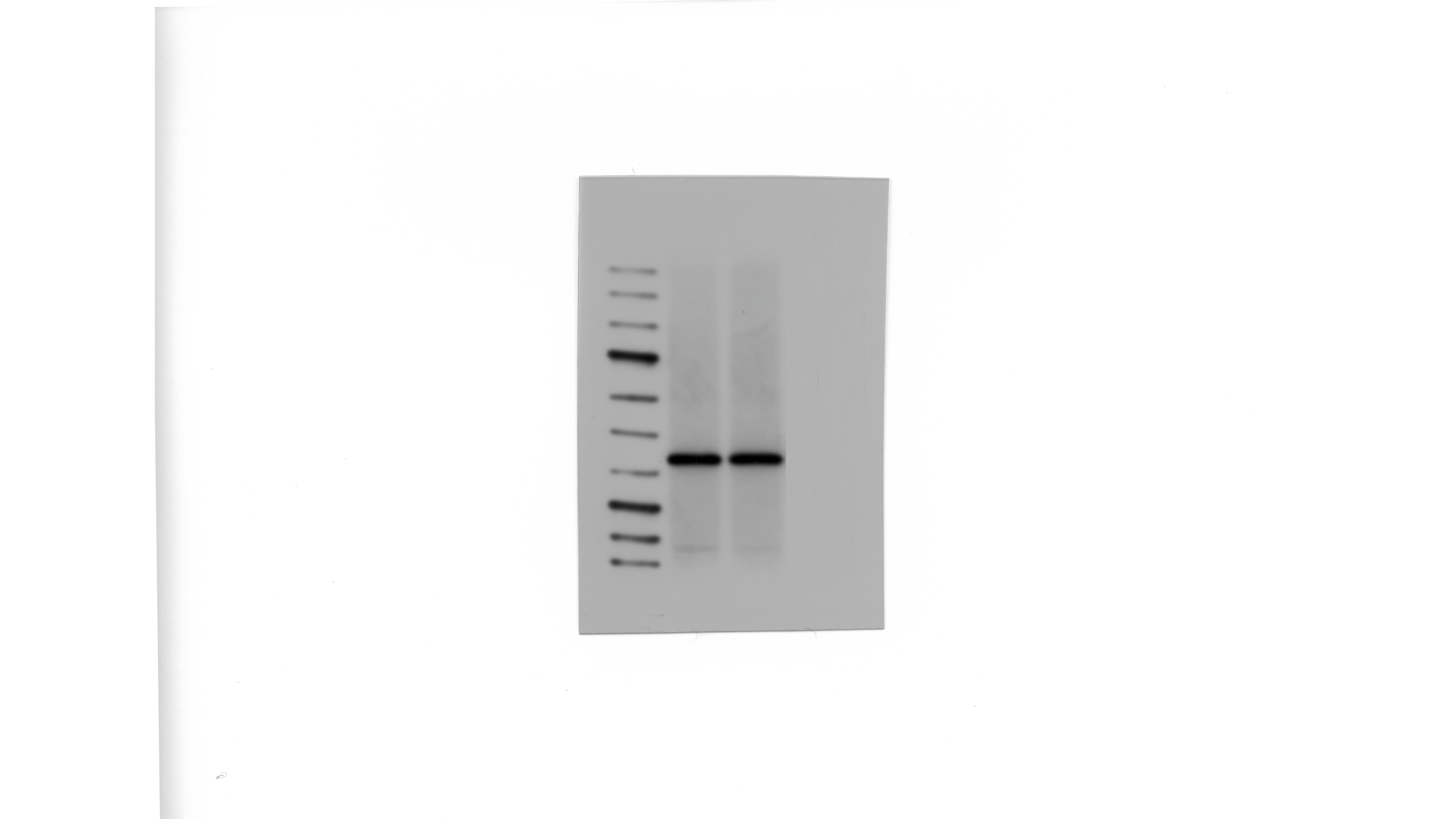

Supplement: Supplementary file 1 — Supplementary Material 1 [file 12884_2025_7983_MOESM1_ESM.zip › Overexpression experiment original WB blot/COX2 GAPDH_600dpi/gapdh_600.tif]

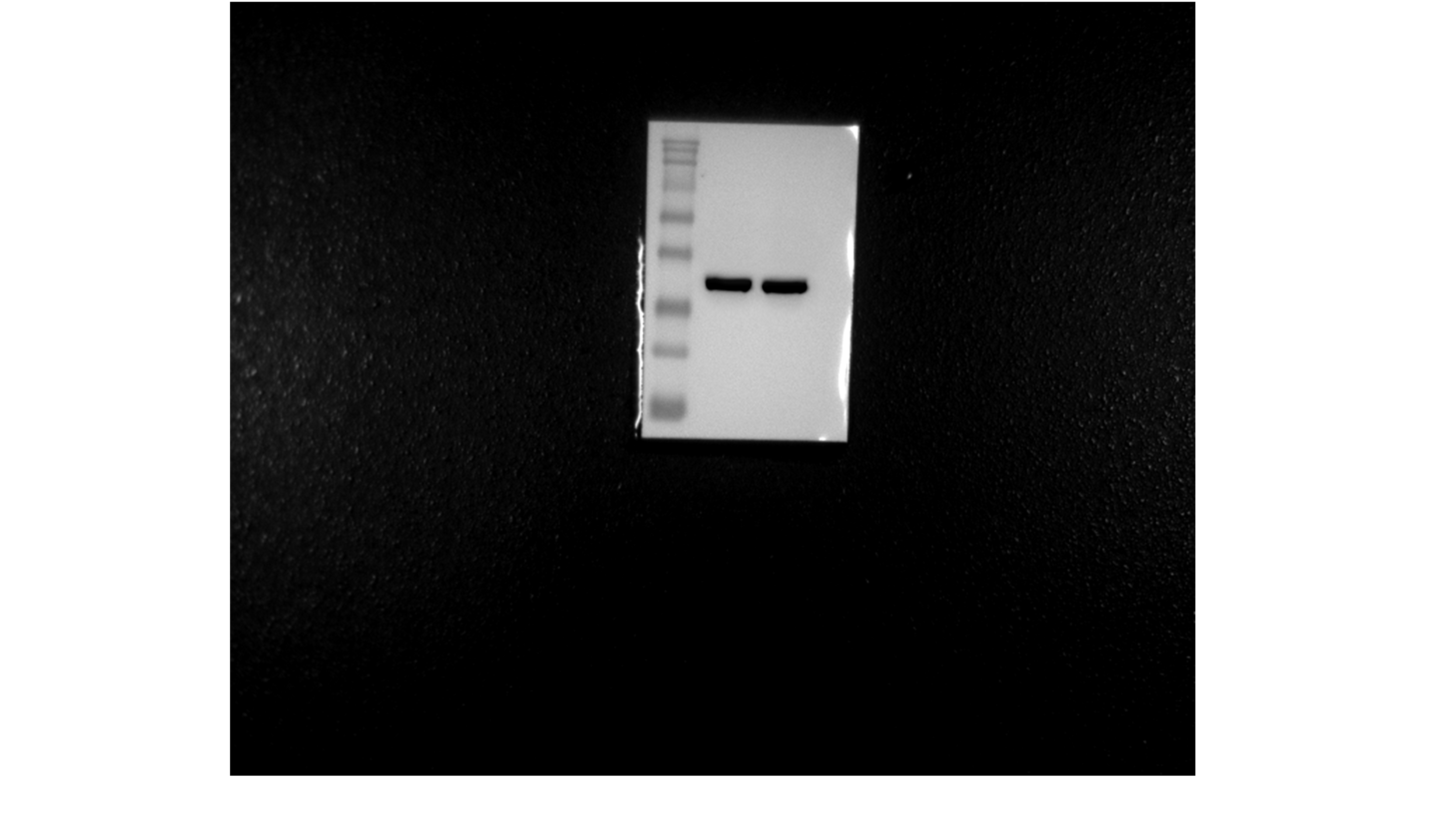

Supplement: Supplementary file 1 — Supplementary Material 1 [file 12884_2025_7983_MOESM1_ESM.zip › Overexpression experiment original WB blot/nf-kb600dpi/GAPDH_600.tif]

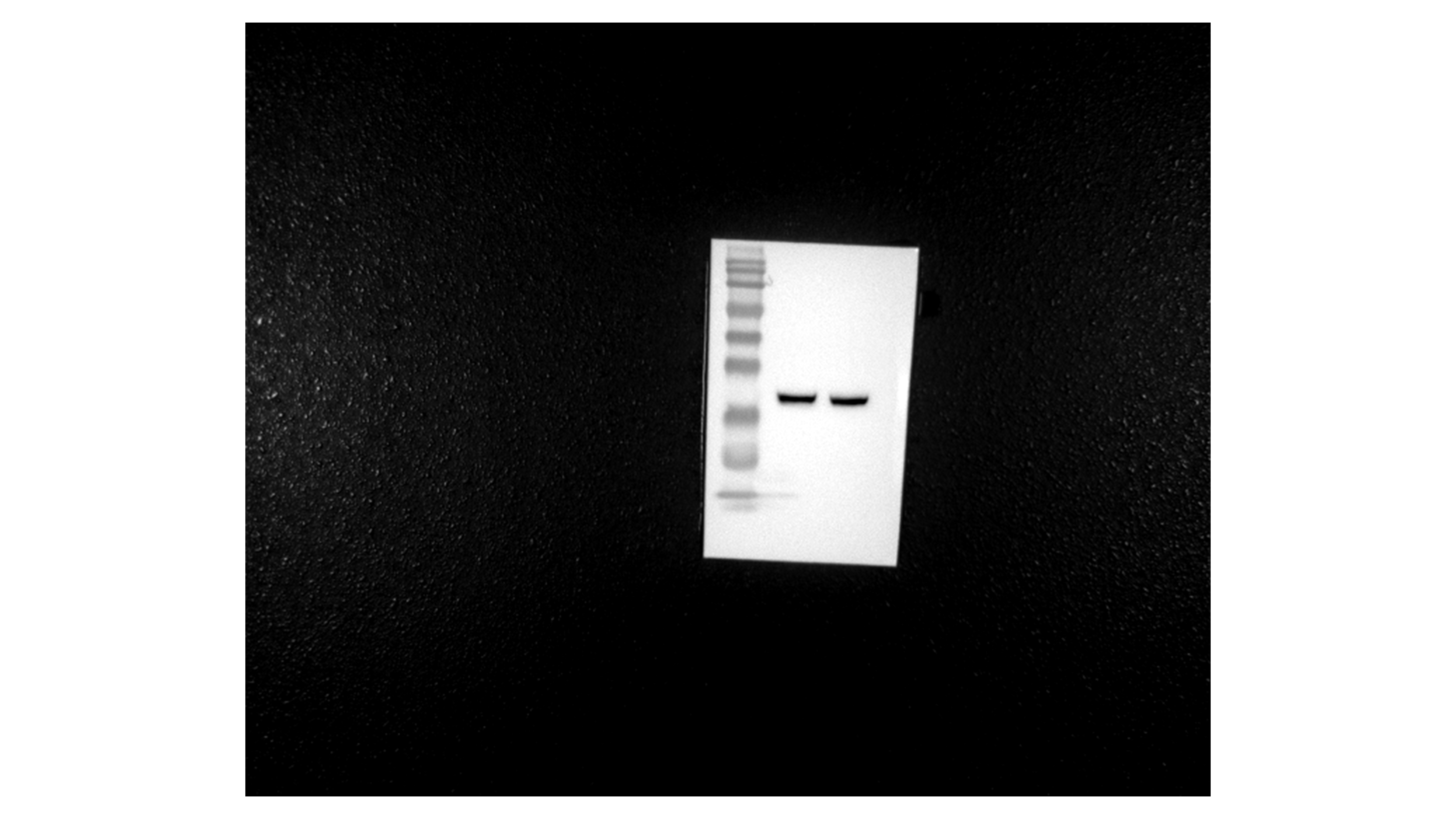

Supplement: Supplementary file 1 — Supplementary Material 1 [file 12884_2025_7983_MOESM1_ESM.zip › Overexpression experiment original WB blot/nf-kb600dpi/IKBa_600.tif]

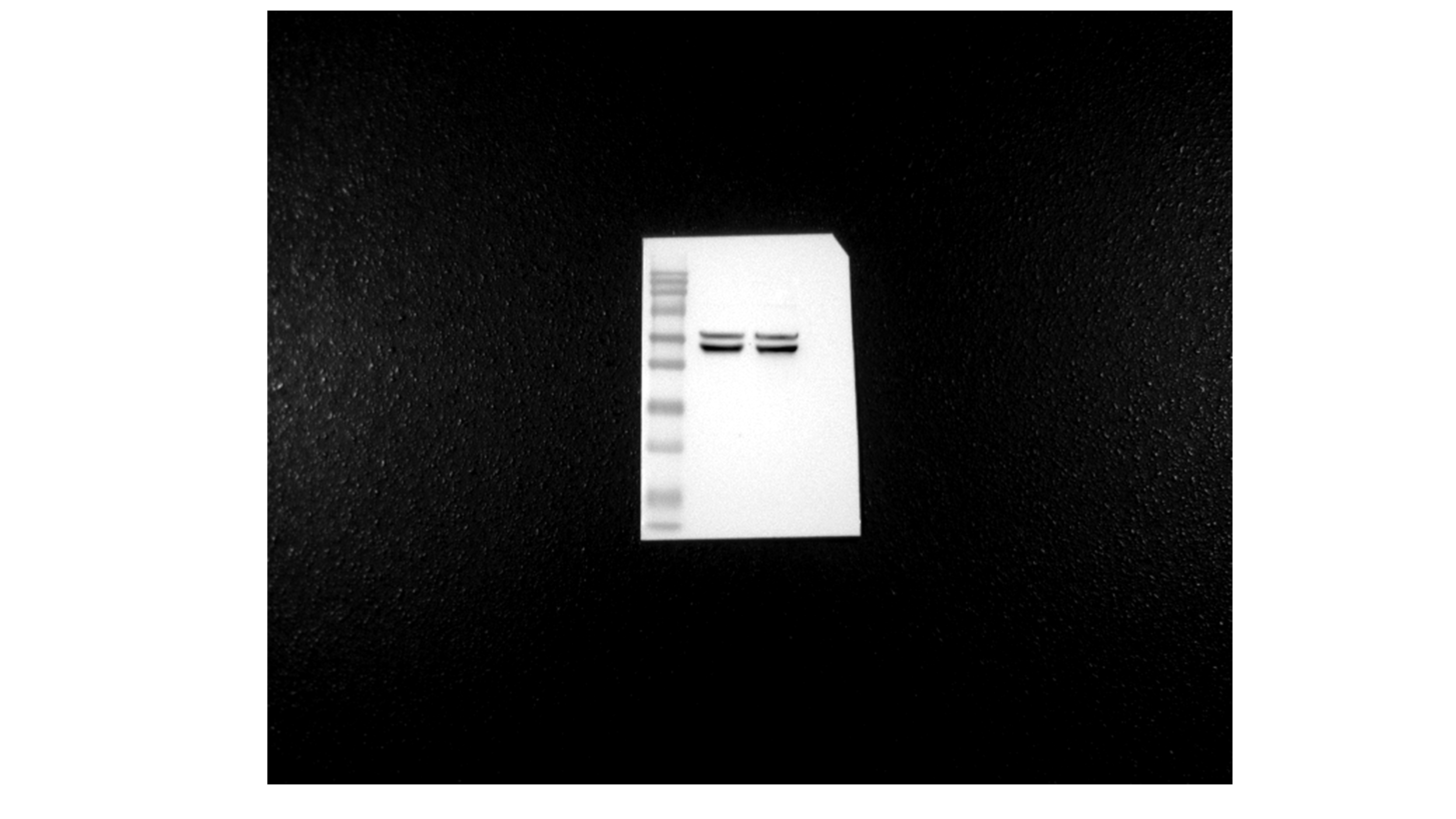

Supplement: Supplementary file 1 — Supplementary Material 1 [file 12884_2025_7983_MOESM1_ESM.zip › Overexpression experiment original WB blot/nf-kb600dpi/JNK_600.tif]

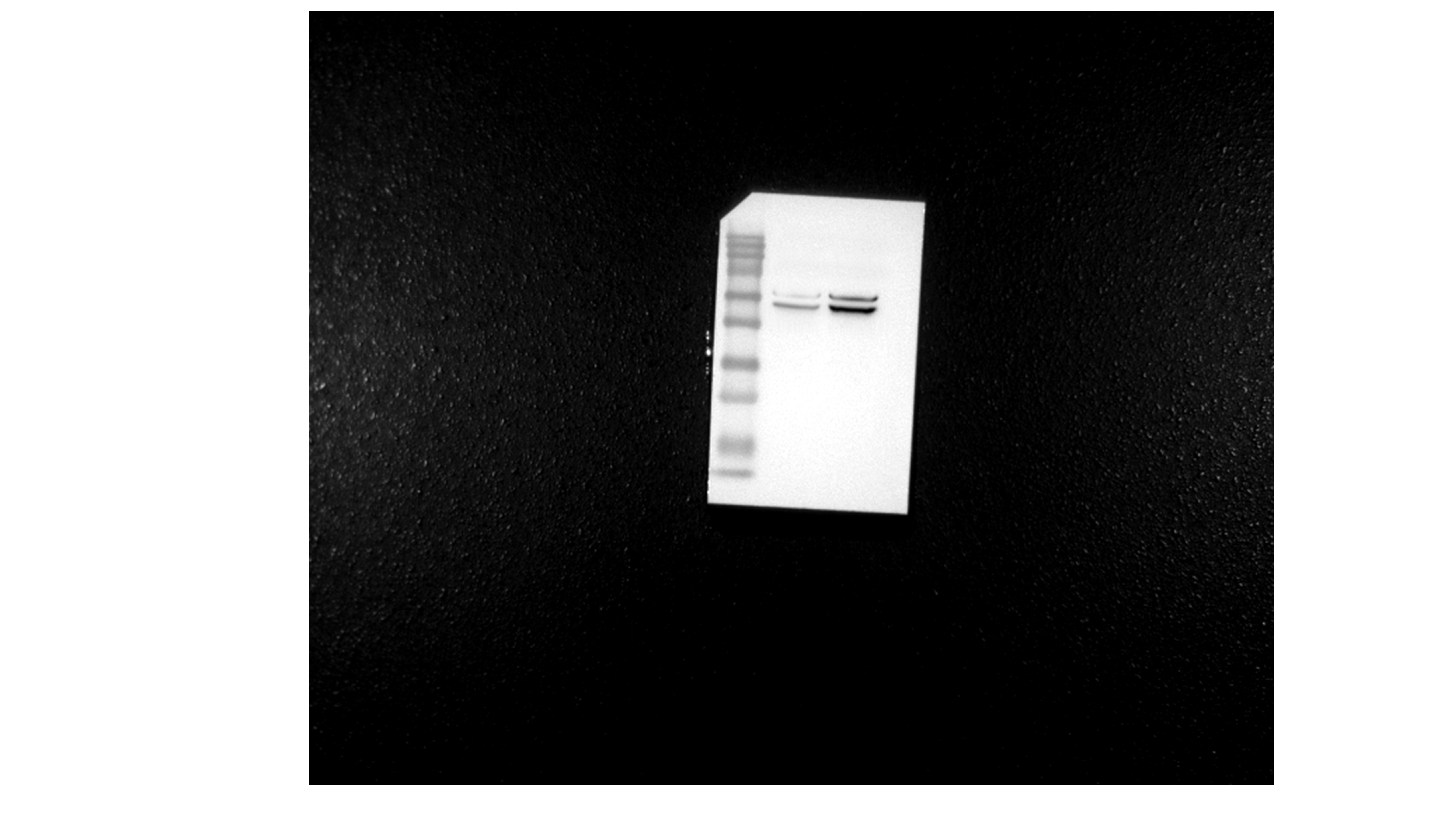

Supplement: Supplementary file 1 — Supplementary Material 1 [file 12884_2025_7983_MOESM1_ESM.zip › Overexpression experiment original WB blot/nf-kb600dpi/p-JNK_600.tif]

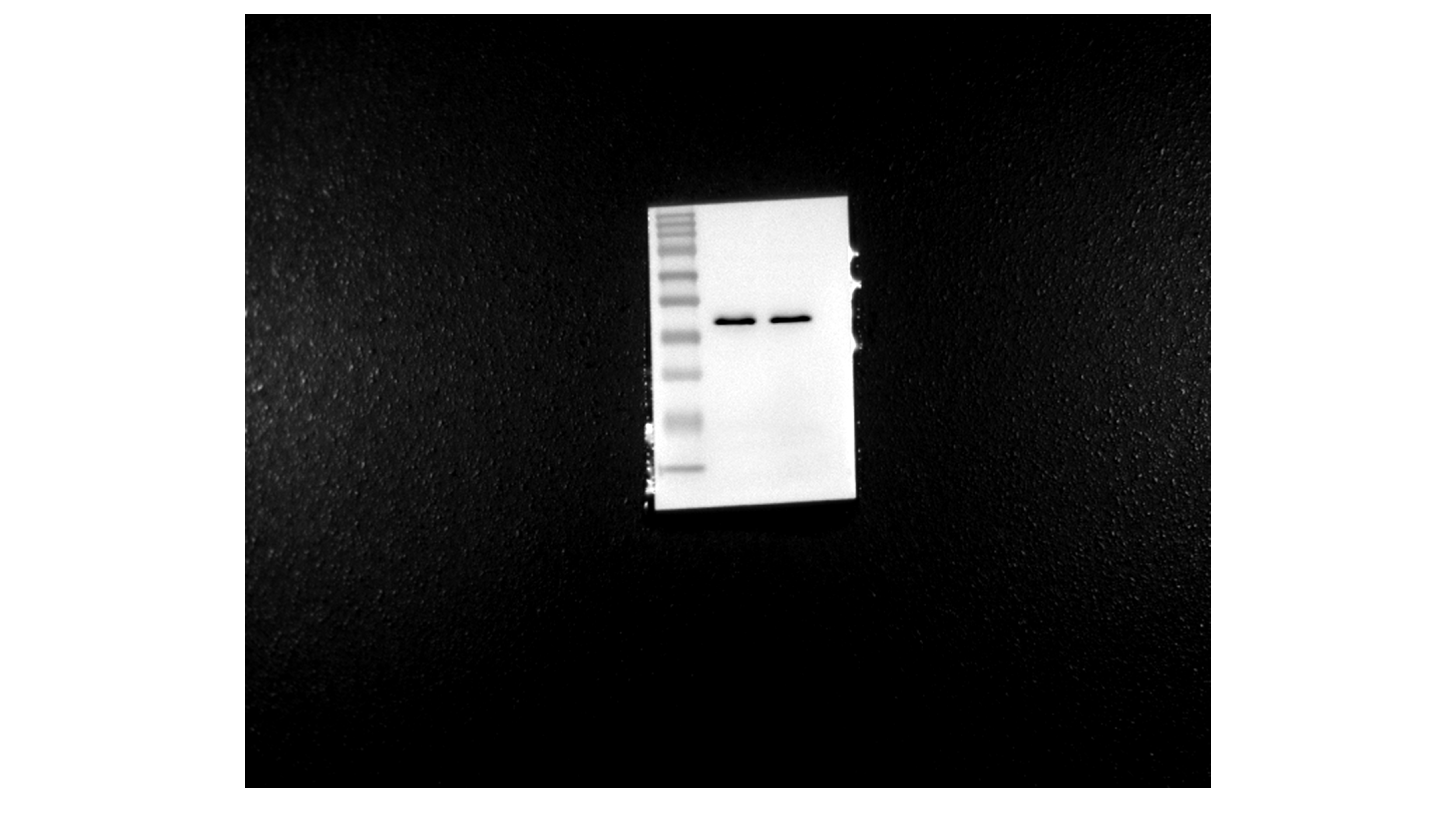

Supplement: Supplementary file 1 — Supplementary Material 1 [file 12884_2025_7983_MOESM1_ESM.zip › Overexpression experiment original WB blot/nf-kb600dpi/P38_600.tif]

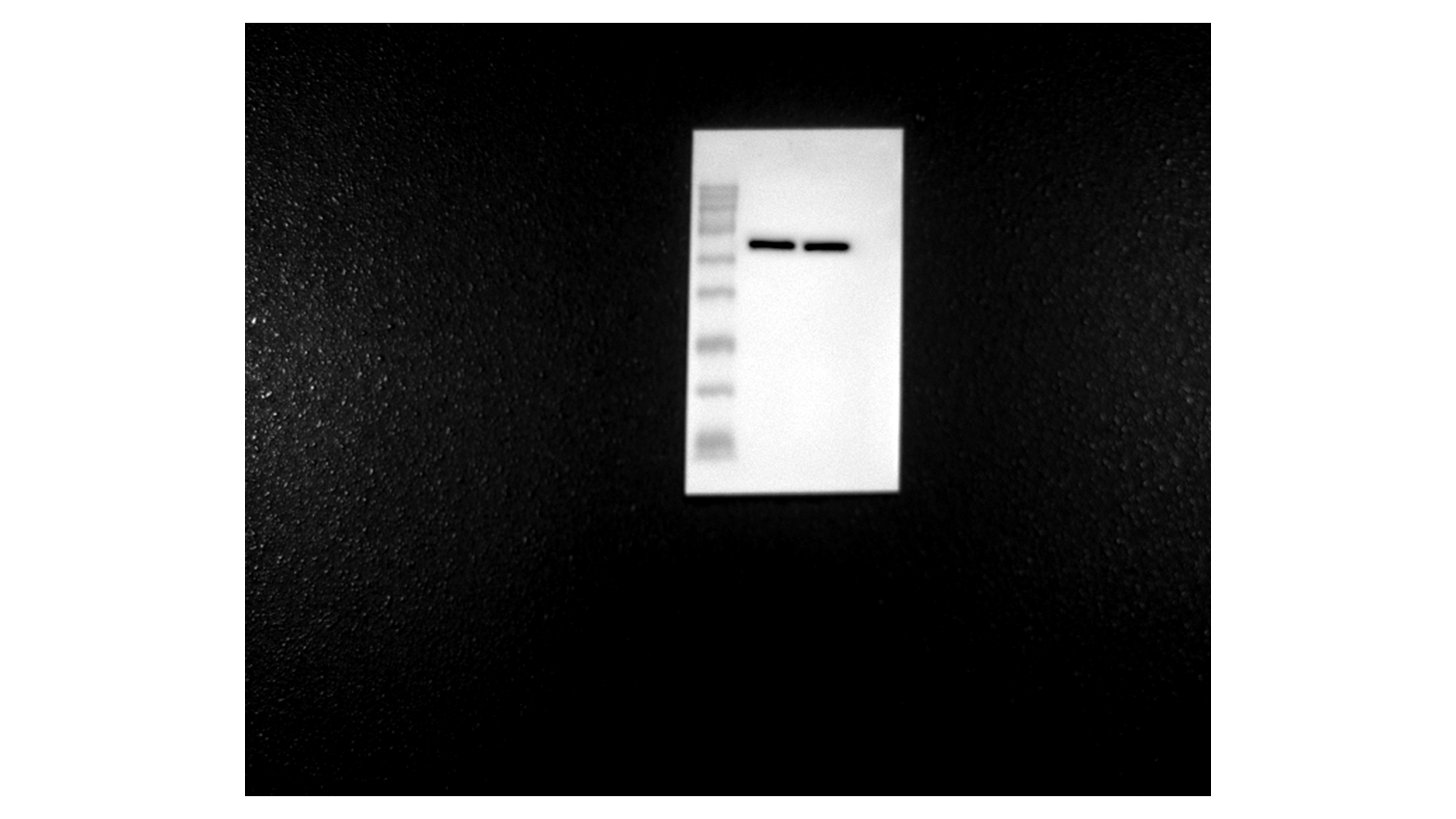

Supplement: Supplementary file 1 — Supplementary Material 1 [file 12884_2025_7983_MOESM1_ESM.zip › Overexpression experiment original WB blot/nf-kb600dpi/P65_600.tif]

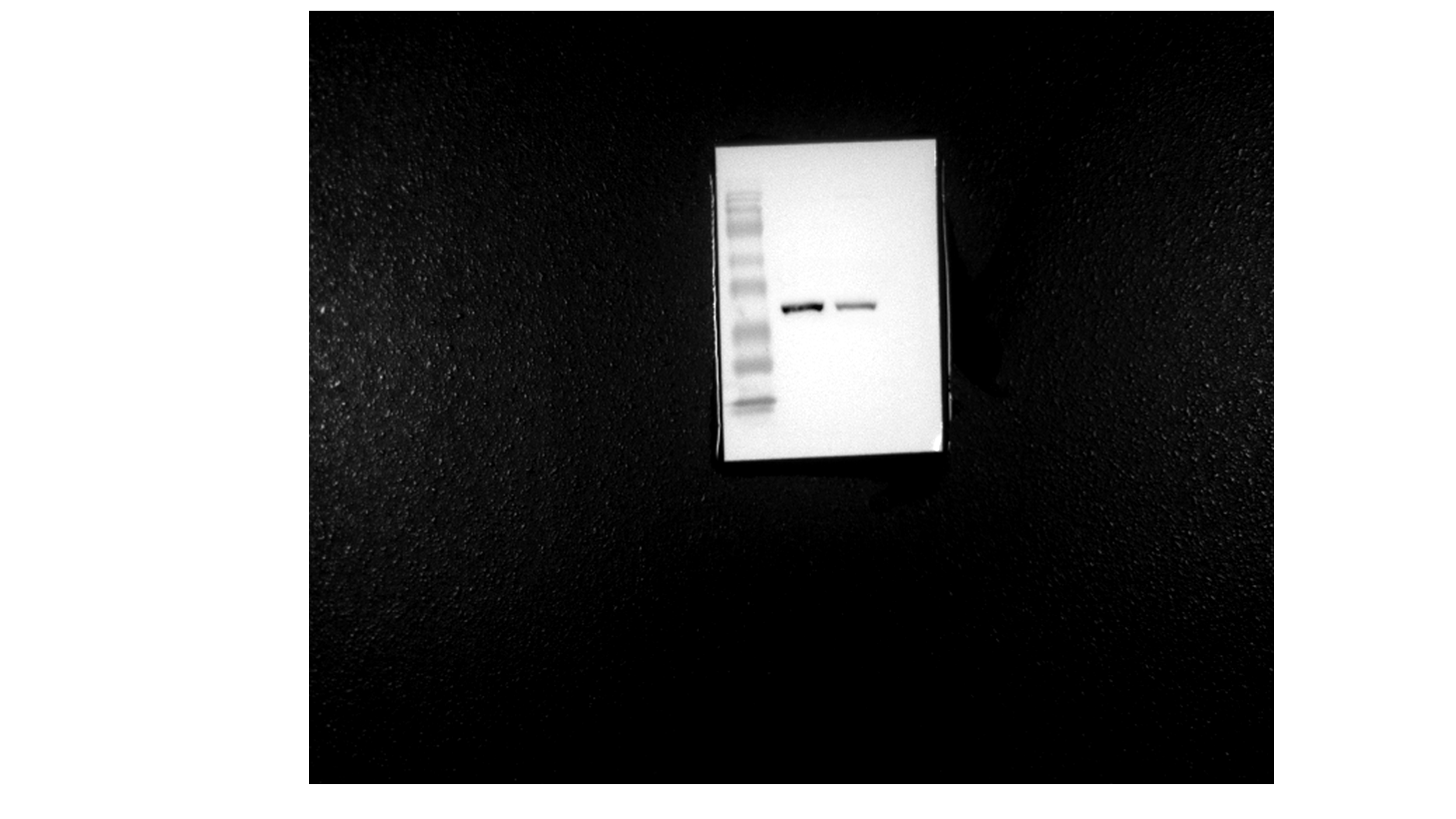

Supplement: Supplementary file 1 — Supplementary Material 1 [file 12884_2025_7983_MOESM1_ESM.zip › Overexpression experiment original WB blot/nf-kb600dpi/pIKBa_600.tif]

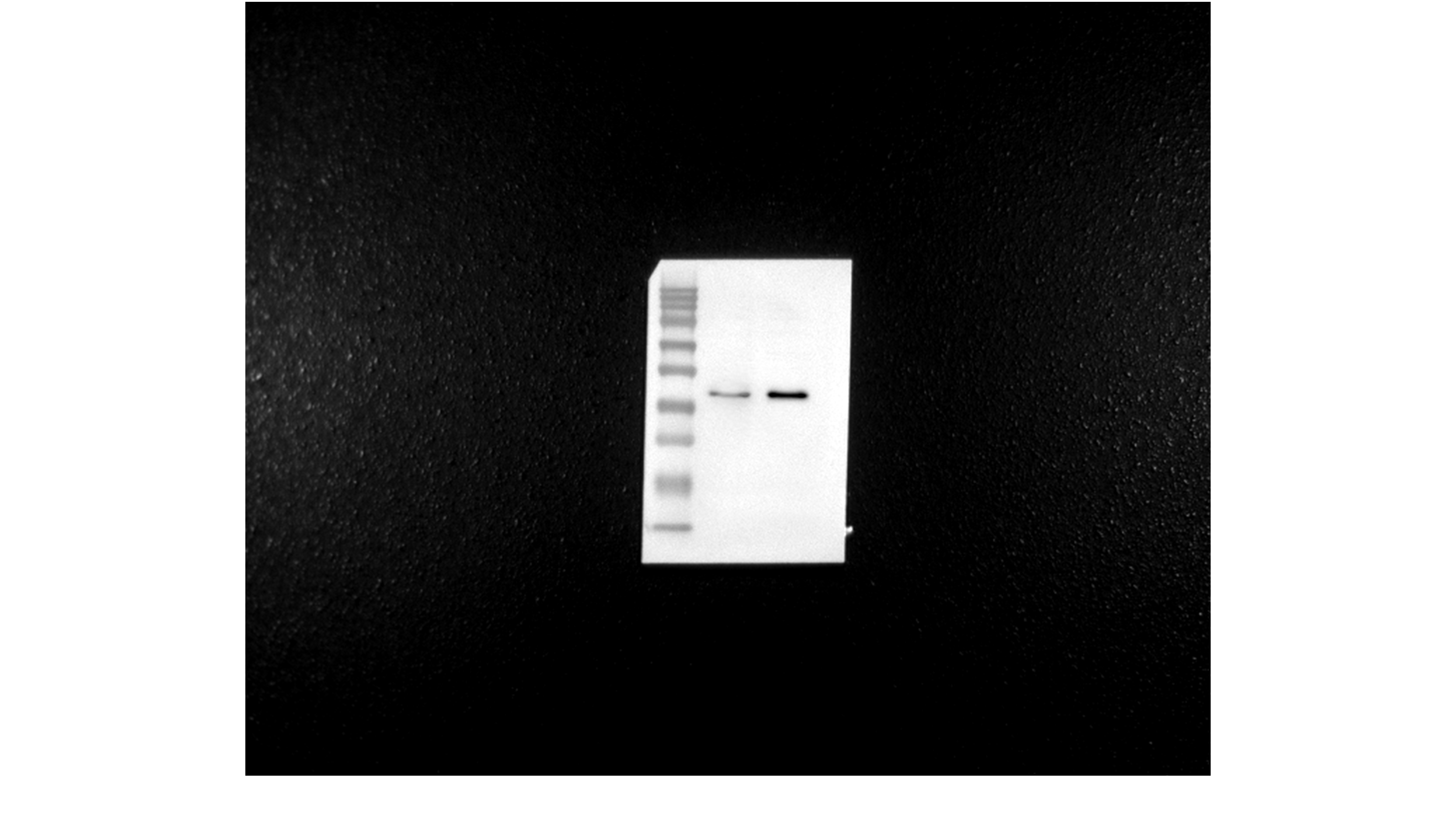

Supplement: Supplementary file 1 — Supplementary Material 1 [file 12884_2025_7983_MOESM1_ESM.zip › Overexpression experiment original WB blot/nf-kb600dpi/PP38_600.tif]

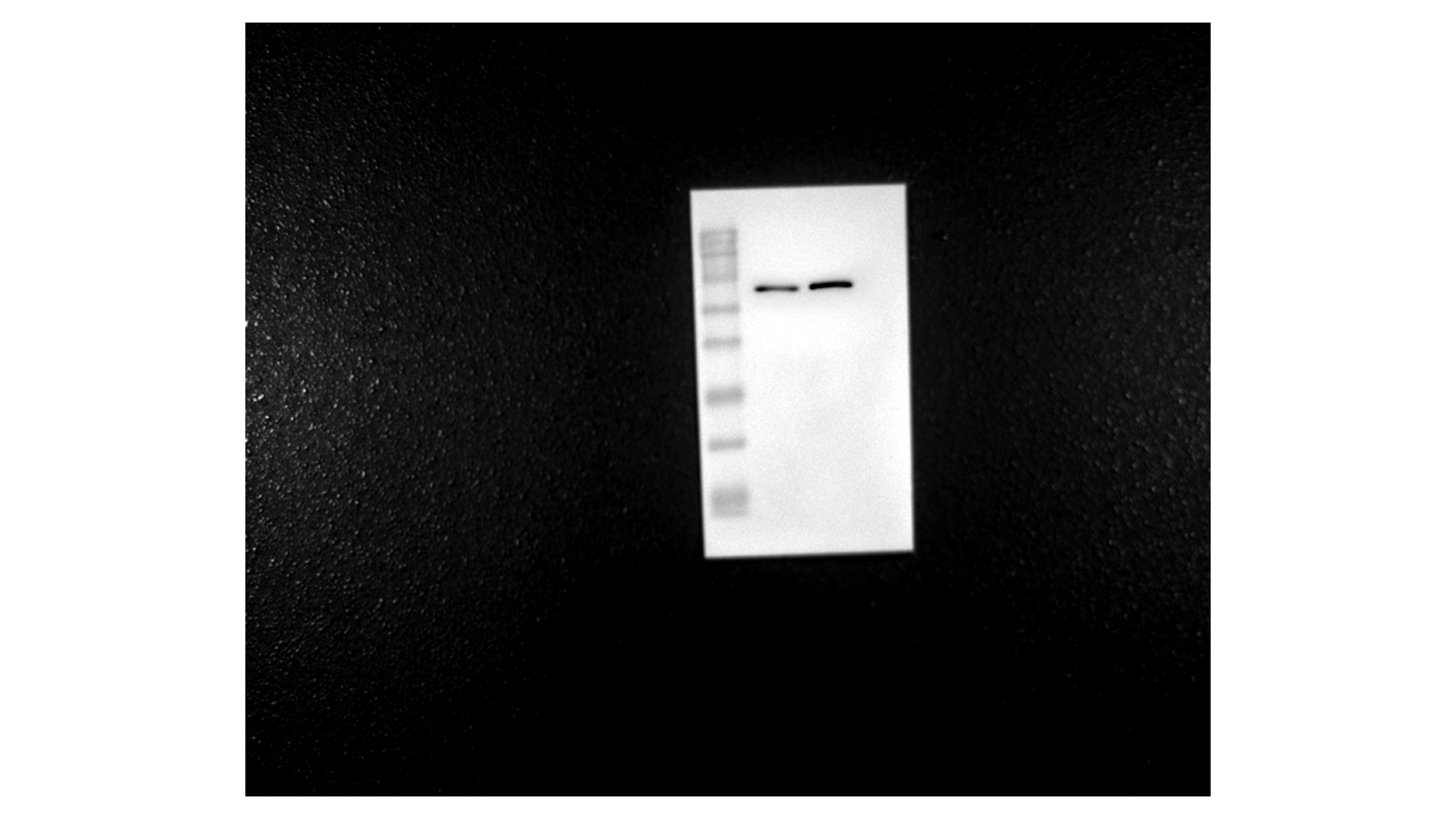

Supplement: Supplementary file 1 — Supplementary Material 1 [file 12884_2025_7983_MOESM1_ESM.zip › Overexpression experiment original WB blot/nf-kb600dpi/PP65_600.tif]
